# Supplementary material for: Revealing a hidden conducting state by manipulating the intracellular domains in KV10.1 exposes the coupling between two gating mechanisms
Source: eLife. 2024 Sep 11;12:RP91420. doi: 10.7554/eLife.91420 (PMC11390113; doi:10.7554/eLife.91420)
Supplement: Supplementary file 1. [file elife-91420-supp1.docx]

|  | ∆2-10 | ∆PASCap | ∆eag | E600R |
| --- | --- | --- | --- | --- |
| A_0_ | -0.052 | -0.054 | -0.067 | -0.091 |
| A_1_ | 1.78 | 1.78 | 1.32 | 2.99 |
| Vh_1_ | -33.57 | -33.57 | -33.57 | -33.57 |
| K_1_ | 16.38 | 16.38 | 16.38 | 16.38 |
| A_2_ | 1.31 | 1.18 | 0.25 | 0.99 |
| Vh_2_ | 51.63 | 79.63 | 102.70 | 83.04 |
| K_2_ | 46.05 | 25.38 | 6.41 | 22.51 |
| Vh_3_ | -33.47 | -5.16 | 55.52 | -21.93 |
| K_3_ | 23.47 | 27.12 | 27.01 | 34.9 |

Table S1. Parameters of a global fit that linked the first component of the biphasic response.
