# Supplementary material for: Revealing a hidden conducting state by manipulating the intracellular domains in KV10.1 exposes the coupling between two gating mechanisms
Source: eLife. 2024 Sep 11;12:RP91420. doi: 10.7554/eLife.91420 (PMC11390113; doi:10.7554/eLife.91420)
Supplement: Supplementary file 2. [file elife-91420-supp2.docx]

|  | K_V_10.1 ∆PASCap | | | | K_V_10.1 E600R | | | | K_V_11.1 | | | |
| --- | --- | --- | --- | --- | --- | --- | --- | --- | --- | --- | --- | --- |
| rate x | x_0_ [s^‑1^] | A_x_  [s^-1^] | V_hx_ [V] | K_x_ [V^-1^] | x_0_ | A_x_  [s^-1^] | V_hx_ [V] | K_x_ [V^-1^] | x_0_ | A_x_  [s^-1^] | V_hx_ [V] | K_x_ [V^-1^] |
| α | 8 | 192 | 0.09 | -55 | 8 | 192 | 0.09 | -55 | 0.5 | 99.5 | 0.09 | -30 |
| β | 4 | 596 | -0.13 | 80 | 4 | 596 | -0.13 | 80 | 1.2 | 16.8 | -0.1 | 80 |
| γ | 5 | 595 | 0.05 | -30 | 5 | 595 | 0.05 | -30 | 2.5 | 297.5 | 0.05 | -30 |
| δ | 15 | 235 | 0.055 | 30 | 15 | 235 | 0.055 | 30 | 7.5 | 117.5 | 0.025 | 30 |
| ε | 200 | 0 | — | — | 200 | 0 | — | — | 200 | 0 | — | — |
| ζ | 660 | 0 | — | — | 1440 | 0 | — | — | 24000 | 0 | — | — |
| η | 80 | 320 | 0 | -80 | 80 | 320 | 0 | -80 | 80 | 320 | 0 | -80 |
| θ | 16 | 44 | 0 | -50 | 16 | 44 | 0 | -50 | 16 | 44 | 0 | -50 |
| κ_R_ | 0.125 | 69.875 | -0.1 | 200 | 0.125 | 69.875 | -0.1 | 200 | 0.65 | 119.35 | -0.1 | 200 |
| κ_L_ | 0.125 | 499.875 | 0.09 | -130 | 0.125 | 499.875 | 0.09 | -130 | 0.65 | 499.35 | 0.09 | -130 |
| λ | 0.1 | 999.9 | -0.12 | 170 | 0.1 | 999.9 | -0.12 | 170 | 0.1 | 999.9 | -0.115 | 180 |
| r_open_ | 100 | 0 | — | — | 30 | 0 | — | — | 30 | 0 | — | — |
| r_close_ | 450 | 0 | — | — | 330 | 0 | — | — | 5970 | 0 | — | — |

**Table S2.** Model parameters.
