# Supplementary material for: Revealing a hidden conducting state by manipulating the intracellular domains in KV10.1 exposes the coupling between two gating mechanisms
Source: eLife. 2024 Sep 11;12:RP91420. doi: 10.7554/eLife.91420 (PMC11390113; doi:10.7554/eLife.91420)
Supplement: Supplementary file 3. — The sequences are listed 5’–3'. For mutagenesis primers, only the sense sequences are given. The reverse primers corresponded to the reverse-complement sequence. [file elife-91420-supp3.docx]

| **Deletion/Mutation** | **Template (s)** | **Primers** |
| --- | --- | --- |
| ∆2-10 | pSGEM.Kv10.1  pSGEM Kv10.1 L341Split | F attcgatatcaagcttatggtggcccctcaaaacacgtttct  R cggtatcgataagcttcagctggctccaaaaatgtctctct |
| ∆PASCap  2-25 | pSGEM Kv10.1 | F gctgccgccaccatg aatgatactaattttgtgttggggaa  R catggtggcggcagctcg |
| ∆CNBHD  525-697 | psGEM Kv10.1. | F tccagaggcattgac aatgaggcccccctgatcttgc  R gtcaatgcctctggacatggaccaa |
| L322H | pSGEM Kv10.1 ∆PASCap pSGEM Kv10.1 E600R | F gatgagggcatcagcagccatttcagctctctaaaagttgt  R acaacttttagagagctgaaatggctgctgatgccctcatc |
| N-terminal CaM binding site (BDN) F151NL154N | pSGEM Kv10.1 ∆2-10  pSGEM Kv10.1 ∆PASCap | F151N ctcttgtcagccgagcattcttcccccagcctttac  L154N tgcttgtcagtgctcttgtattccgagcattcttcccccag |
| C-terminal CaM binding site (BDC2) F714SF717S | pSGEM Kv10.1 ∆2-10  pSGEM Kv10.1 ∆PASCap | F714S gtcggaatctctggctgaggcgccggacag  F717S ctgctgtcggcttctctggctgaggcgccgg |

**Table S3. Primers used for infusion cloning or site-directed mutagenesis.** The sequences are listed 5’-3'. For mutagenesis primers, only the sense sequences are given. The reverse primers corresponded to the reverse-complement sequence.
